# Efficacy of clozapine for long‐stay patients with treatment‐resistant schizophrenia: 4‐year observational study

**DOI:** 10.1002/npr2.12244

**Published:** 2022-03-02

**Authors:** Kentaro Mizuno, Emiko Mizuno, Akira Suekane, Toshiaki Shiratsuchi

**Affiliations:** ^1^ Wakakusa Hospital Miyazaki Japan; ^2^ Emergency Department Miyazaki Prefectual Miyazaki Hospital Miyazaki Japan

**Keywords:** clozapine, drug therapy, hospitalization, schizophrenia, treatment outcome

## Abstract

**Aim:**

Supporting patients upon discharge following prolonged hospitalization in private psychiatric hospitals in Japan have long been an issue. This study evaluated the efficacy of clozapine in treating long‐stay patients with treatment‐resistant schizophrenia to reduce the frequency and duration of readmissions postdischarge.

**Methods:**

We retrospectively examined the length and frequency of hospitalizations of long‐stay and non‐long‐stay patients with schizophrenia who were introduced to clozapine at our hospital.

**Results:**

Comparing participants’ medical records 2 years before and after the introduction of clozapine, we identified a significant decrease in both length and frequency of hospitalizations in all patients who were introduced to clozapine. In long‐stay patients, the length and frequency of hospitalization were significantly reduced. However, compared to non‐long‐stay patients, the period from introduction of clozapine to discharge was longer and the dose of clozapine was higher. Thus, it is necessary to take time to evaluate the therapeutic effects for long‐stay patients.

**Conclusion:**

The results showed that clozapine is helpful for patients with treatment‐resistant schizophrenia, who are considered difficult to treat and discharge in Japan.

## Abstract 2

We retrospectively examined the length and frequency of hospitalizations of long‐stay and non‐long‐stay patients with schizophrenia who were introduced to clozapine at our hospital. Two years before and after the introduction of clozapine, we identified a significant decrease in both length and frequency of hospitalizations in all patients who were introduced to clozapine.

## Graphical abstract

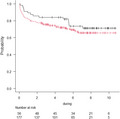

## 1 INTRODUCTION

Clozapine (CLZ) is an antipsychotic drug that has been recommended for use in the treatment of treatment‐resistant schizophrenia (TRS) since 1988, when it was proved to be effective.^1^ In Japan, CLZ has been covered by insurance since July 2009. CLZ has been regarded as an important therapeutic tool to facilitate the transition of TRS patients from hospital settings to the community in Japan.^2^ The Ministry of Health, Labour and Welfare has set targets for infrastructure development based on the demand for hospitalization in psychiatric facilities and the degree of success in community transition. The Ministry aims to increase the prescription rate of CLZ from 25% to 30% for TRS patients by 2025.^3^ However, as of May 28, 2021, the total number of patients registered on the Clozaril Patient Monitoring Service was 12 215; although this figure is gradually increasing, the number of patients registered each year has only reached 2000. In addition, the treatment adherence rate is approximately 70%.^4^ Assuming that there are 200 000 TRS patients in Japan, the rate of prescription has not yet reached 5%.^5^, ^6^ Thus, although measures to promote the use of CLZ have been adopted in recent medical plans and medical fee revisions,^7^, ^8^ it remains a challenge to reach the targets set by the Ministry of Health, Labour and Welfare. The reasons for the lack of widespread introduction of CLZ in Japan relate to concern about blood disorders such as leukopenia and agranulocytosis, and the short intervals between blood tests compared with protocols overseas, which place a burden on both medical personnel and patients. However, the frequency of leukopenia was found to be highest during the first 18 weeks following the initiation of CLZ. Additionally, over 90% of leukopenia cases occurred within 1 year of introduction of CLZ, indicating that there is little need to continue worrying about blood disorders during the maintenance phase of treatment.^3^ Furthermore, blood test intervals have been revised to be similar to those overseas from June 2021; patients are now able to take CLZ with a test interval of 4 weeks if they meet the conditions post 52 weeks following the introduction of CLZ. Despite a gradual lowering of hurdles regarding CLZ prescription and usage, the reality remains that the target is not being reached. This may be due in part to the hesitancy surrounding its use following the results of a recently reported meta‐analysis^9^ based on randomized control trials (RCTs) conducted overseas, which questioned the superiority of CLZ. Therefore, it is important to correctly evaluate the usefulness of CLZ in Japan to promote its prescription and use.

The introduction of CLZ is not widespread, even though there are numerous patients with schizophrenia in Japan who have been hospitalized for 1 year or longer (long‐stay patients). Long‐stay patients not only have chronic and entrenched symptoms but also exhibit a decline in daily living and social skills due to long‐term hospitalization. Accordingly, the patients tend to lose confidence and motivation to leave the hospital. In addition, it is difficult for patients to live in the community after being discharged, and they often fall into a “revolving door phenomenon” of repeated readmissions due to relapse. Both the patient and the medical personnel may have lowered motivation for the new pharmacotherapy.　Therefore, there may be cases in which CLZ cannot be introduced as an active treatment for long‐stay patients. Since December 2009, our institutional policy has ensured that we actively explain the introduction of CLZ to patients and their families who meet the criteria for TRS and introduce CLZ to patients who give their consent. As a result, the ratio of the number of patients hospitalized for more than 1 year to the total number of hospital beds has decreased from approximately 40% to approximately 10% over the past 10 years, and the number of hospital beds has been reduced by 40%, as reported previously.^10^ We have gained extensive experience in using the drug with long‐stay patients, and although discharge coordination was often difficult and required a substantial amount of time and manpower, fewer cases of readmission were observed among the discharged patients. The purpose of this study was to demonstrate the effectiveness of CLZ in reducing the length of hospital stay and prevent re‐hospitalization by comparing the number of hospital stays before and after the introduction of CLZ.

## 2 METHODS

### 2.1 Sample

To be included in the study, patients had to have been observed for 2 years before the introduction of CLZ, for 18 weeks after the start of CLZ treatment, and for 2 years thereafter. Therefore, 286 patients who enrolled in the Clozaril Patient Monitoring Service from December 28, 2009, to May 27, 2018, were included in the study. All participants had been diagnosed with TRS and undertook CLZ treatment. Of these, 28 re‐enrolled patients and 25 patients for whom follow‐up was not possible after introduction were excluded, and 233 patients were included in the statistical analysis. These 233 patients were divided into two groups, 55 in the long‐stay group and 178 in the non‐long‐stay group, and their data were compared. The definitions of the long‐stay and non‐long‐stay groups were as follows: Patients who had been hospitalized for more than 1 year in the 2 years prior to the introduction of CLZ were included in the long‐stay group, and the remaining patients were included in the non‐long‐stay group. In Japan, there are 170 000 long‐term inpatients who have been hospitalized for more than 1 year, of which difficulties with discharge are experienced in 30% owing to a lack of residential and support systems, 10% because of physical complications from treatment, and the remaining 60% owing to extremely severe or unstable psychiatric symptoms. Although CLZ is expected to be widely used as a treatment for TRS, there are no reports of its use in long‐term hospitalized patients, and the usefulness of CLZ for long‐term hospitalized patients has not yet been demonstrated. However, we hold that CLZ should be introduced to improve psychiatric symptoms in long‐stay hospitalized patients. In this study, we investigated the effects of CLZ on shortening the length of hospitalization and preventing re‐hospitalization in long‐stay hospitalized patients.

### 2.2 Definition of periods

The preintroduction period was defined as 2 years before the beginning of CLZ introduction, and the postintroduction period was defined as 2 years from 18 weeks following CLZ introduction. These 18 weeks were regarded as the base period, in consideration of the principle that CLZ treatment requires hospitalization for 18 weeks in Japan and a withdrawal period from any previous drugs. The 1st year of the preintroduction period was defined as Year 1, the 2nd year of the preintroduction period (up to the day before the start of introduction) as Year 2, the period from 18 weeks following the start of CLZ introduction to 1 year after the start of introduction as Year 3, and the 1 year following Year 3 as Year 4.

### 2.3 Ethical considerations

This study was approved by the ethics committee of the Wakakusa Hospital (approval number 2020‐001). Medical records of eligible patients from December 28, 2007, to September 30, 2020, were analyzed. The purpose of this study and the process to request exclusion were posted in the hospital, and an adequate opt‐out period was provided. Data were anonymized such that individuals could not be identified.

### 2.4 Research design

**FIGURE 1:**
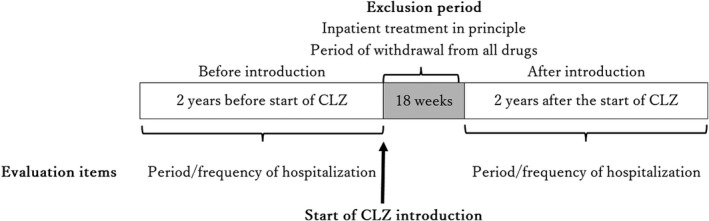
Period for evaluation of effectiveness. The preintroduction period was defined as 2 years before the beginning of CLZ treatment, and the postintroduction period was defined as 2 years from the 18 weeks following CLZ introduction. The 1st year of the preintroduction period was defined as Year 1; the 2nd year of the preintroduction period (up to the day before the start of introduction) was defined as Year 2; the period from 18 weeks following the start of CLZ introduction to 1 year after the start of introduction was defined as Year 3, and the 1 year following Year 3 was defined as Year 4

This mirror‐image study retrospectively examined changes in both the length and number of hospitalizations over a period of 2 years before and after the introduction of CLZ. The effects of CLZ were compared between the long‐stay and non‐long‐stay groups (Figure 1).

### 2.5 Data collection

In terms of demographic and background details, we collected data on gender, duration of illness, age at the start of treatment, reason(s) for introduction of CLZ, and dosage of antipsychotic drugs before introduction of CLZ. The Clinical Global Impression Scale‐Severity (CGI‐S) was administered at the time of introduction of CLZ and at the time of discharge. The CGI‐S is a 7‐point scale that requires the clinician to rate the severity of a patient's illness at the time of assessment, relative to the clinician's past experience with patients who have the same diagnosis (1. normal, 2. borderline, 3. mildly ill, 4. moderately ill, 5. markedly ill, 6. severely ill, and 7. most extremely ill).^11^ CLZ dose at the time of discharge or at the end of the study was recorded along with the number of days between the introduction of CLZ and discharge, and the duration of CLZ administration. Details regarding CLZ treatment adherence, the length of stay, and the frequency of readmission in the long‐stay and non‐long‐stay groups were also recorded.

### 2.6 Data analysis

The data were analyzed using EZR version 1.54.^12^ The data were tested for normality, and it was found that the duration of disorder, age, CLZ dose and dosing period, CGI‐S, duration of hospitalization until discharge, length of hospitalization before and after introduction of CLZ, and number of instances of hospitalization were not normally distributed. Accordingly, the Mann–Whitney U test was used to compare the long‐stay group with the non‐long‐stay group on these variables. The estimated CLZ dose adherence rates were calculated using log‐rank tests. The Wilcoxon signed‐rank test was used to compare the length and frequency of hospitalizations before and after the introduction of CLZ. The significance value was set at 0.05.

## 3 RESULTS

### 3.1 Sample characteristics

Table 1 shows the background details of the full sample, and the long‐stay and non‐long‐stay groups. The total number of patients surveyed was 233 (117 male, 116 female). The median age at the time of CLZ introduction was 43 years, the median illness duration was 20 years, the median chlorpromazine‐equivalent antipsychotic dose before CLZ introduction was 800 mg, the median CGI‐S at time of CLZ introduction was 6, and the median duration of CLZ treatment was 1925 days. A total of 230 patients (98.7%) were discharged during the study period; in terms of median values of the entire group, the CLZ dose at first discharge was 200 mg, and the duration of hospitalization from CLZ introduction to discharge was 124.5 days. The frequency of hospitalization for 2 years after CLZ introduction was 1, and CGI‐S at discharge was 4. In a comparison between the long‐stay and non‐long‐stay groups, there were no significant differences in age at introduction, sex, illness duration and antipsychotic dose before the introduction, frequency of hospitalization for 2 years after CLZ introduction, CGI‐S before introduction, adherence rate, or CGI‐S at discharge. However, there were significant differences in the duration of CLZ administration, length of hospitalization in the 2 years before introduction, CLZ dose at discharge, and duration of hospitalization between introduction and discharge. The median duration of CLZ treatment was 2696 days in the long‐stay group versus 1722 days in the non‐long‐stay group, with a significantly longer duration for the long‐stay group (*P* < .0001). The median length of hospital stay in the long‐stay group was 665 days, whereas it was 108 days in the non‐long‐stay group, signifying a significantly longer period for the long‐stay group (*P* < .0001). The median CLZ dose at the time of discharge was 300 mg in the long‐stay group versus 200 mg in the non‐long‐stay group, with the long‐stay group receiving a significantly higher dose (*P* < .001). The median time from the introduction of CLZ to hospital discharge was 215 days in the long‐stay group and 101 days in the non‐long‐stay group, indicating a significantly longer time for the long‐stay group (*P* < .0001).

**TABLE 1:** Patient background data

Background Information	All subjects (n = 233)	Long‐stay group (n = 55)	Non‐long‐stay group (n = 178)	*P* value
No. of males (%)	117 (50.2%)	32 (57.1%)	85 (48.0%)	0.236
Age at introduction	43 (33, 51)	43.5 (32.75, 54)	42 (34, 50)	0.785
Duration of illness (years)	20 (12, 28)	22 (12.75, 31)	20 (12, 26)	0.136
Antipsychotic dose before CLZ (mg: CPZ equivalent)	800 (600, 900)	800 (600, 868.75)	800 (600, 900)	0.856
CGI‐S before introduction of CLZ	6 (6, 6)	6 (6, 6)	6 (6, 6)	0.066
Duration of CLZ treatment (days)	1925 (949, 2765)	2696 (1832.75, 3179.75)	1722 (909, 2562)	<0.0001**
Length of hospitalization in the 2 years prior to CLZ introduction (days)	163 (68, 349)	665 (489.25, 730)	108 (44, 190)	<0.0001**
Adherence rate (%)	70.4	73.2	69.5	0.597

Information at discharge	All subjects (n = 230)	Long‐stay group (n = 54)	Non‐long‐stay group (n = 176)	*P* value
CLZ dose at discharge (mg)	200 (100, 300)	300 (200, 350)	200 (100, 300)	<0.001**
Length of hospitalization from introduction of CLZ to discharge (days)	124.5 (76.25, 196)	215.5 (146.25, 494.25)	101 (69.75, 155.5)	<0.0001**
Frequency of hospitalization	1 (0, 2)	1 (0, 2)	1 (0, 2)	0.445
CGI‐S at discharge	4 (4, 4)	4 (4, 5)	4 (4, 4)	0.111

Note

Median (lower quartile and upper quartile).

**P < .01.

### 3.2 Treatment adherence rate

**FIGURE 2:**
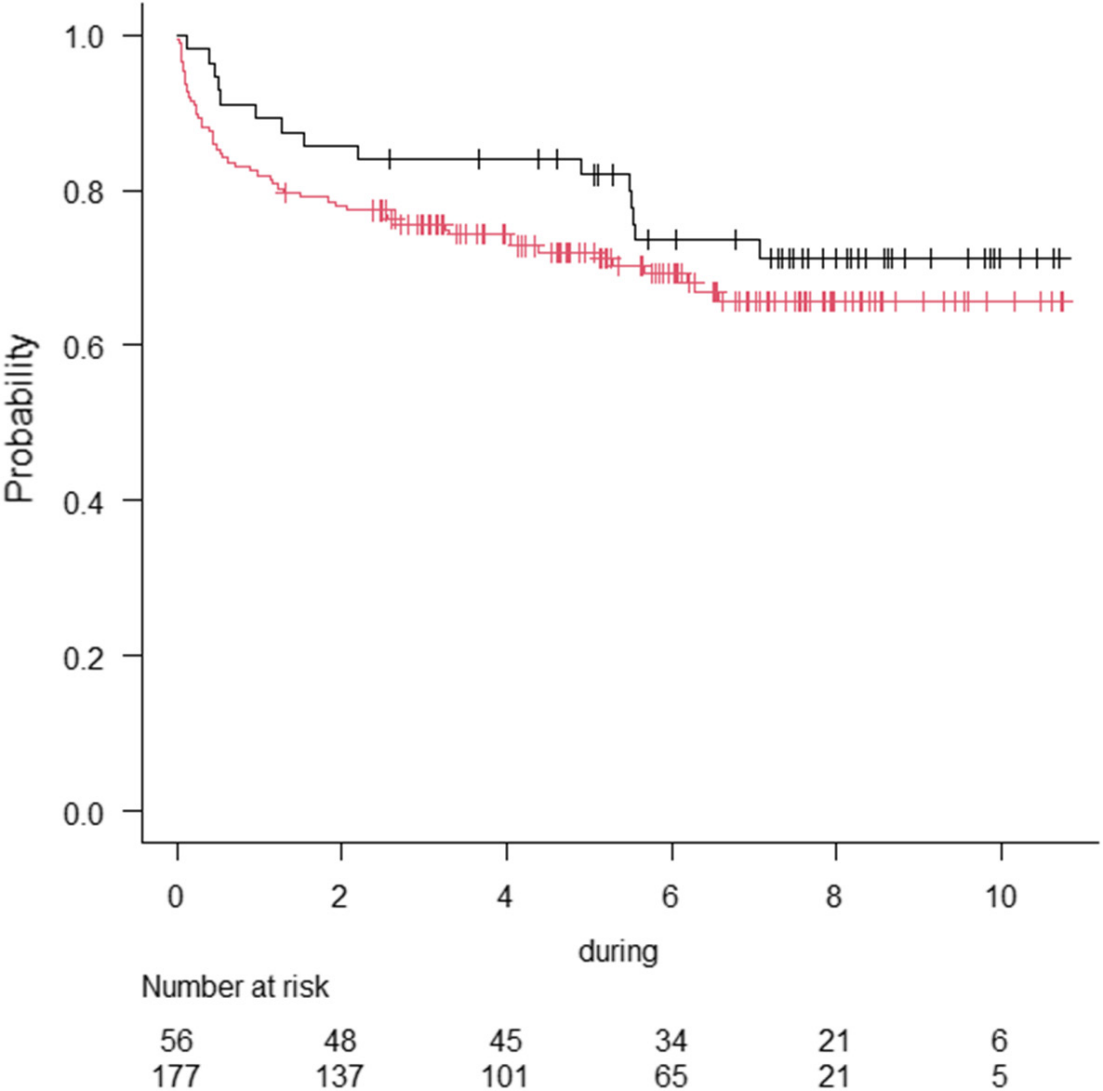
Estimated CLZ treatment adherence rate. Clozapine treatment continuation rates among patients. A Kaplan–Meier survival plot for the rate of clozapine treatment continuation. Lines represent the long‐stay group (—) and the non‐long‐stay group (—)

The estimated CLZ treatment adherence rates in the long‐stay and non‐long‐stay groups are shown in Figure 2. A total of 69 patients (29.6%) discontinued CLZ treatment after introduction. The overall estimated treatment adherence rate was 83.3% (95%, CI: 77.8%‐87.5%) at 1 year, 79.4% (95%, CI: 73.6%–84.0%) at 2 years, and 73.9% (95%, CI: 67.5%‐79.1%) at 5 years. The estimated treatment adherence rate was not significantly different in the long‐stay and non‐long‐stay groups (*P* = .278), and the estimated adherence rate at 2 years was 83.9% in the long‐stay group and 77.4% in the non‐long‐stay group.

### 3.3 Comparison of the length and frequency of hospitalizations before and after the introduction of CLZ in the long‐stay and non‐long‐stay groups

The length of hospitalization at years 1, 2, 3, 4, and 18 weeks after the start of the introduction is shown in Table 2. Figure 1 shows the length of hospitalization of the whole sample, the long‐stay group, and the non‐long‐stay group in Years 1 and 2 (before the introduction of CLZ), Years 3 and 4 (after the introduction of CLZ) and during the base period. The frequency of hospitalization was also compared in the same manner. Table 3 shows the length and frequency of hospitalizations of the whole sample, and of the long‐stay and non‐long‐stay groups, before and after the introduction of CLZ.

**TABLE 2:** Length of hospitalization in each period

	All subjects (n = 233)	Long‐stay group (n = 55)	Non‐long‐stay group (n = 178)
Year 1			
Length of hospitalization (days)	47 (0, 189)	365 (250.5, 365)	11 (0, 74)
Frequency of hospitalization	0 (0, 1)	0 (0, 1)	0 (0, 1)
Year 2			
Length of hospitalization (days)	100 (34, 213)	365 (280.5, 365)	67 (17, 123)
Frequency of hospitalization	1 (1, 2)	1 (0, 1)	1 (1, 2)
18 weeks after start			
Length of hospitalization (days)	126 (78, 126)	126 (126, 126)	103.5 (72, 126)
Frequency of hospitalization	0 (0, 0)	0 (0, 0)	0 (0, 0)
Year 3			
Length of hospitalization (days)	62 (0, 154)	165 (78.5, 365)	39 (0, 95)
Frequency of hospitalization	0 (0, 1)	0 (0, 1)	0 (0, 1)
Year 4			
Length of hospitalization (days)	0 (0, 85)	77 (0, 212)	0 (0, 53.75)
Frequency of hospitalization	0 (0, 1)	0 (0, 1)	0 (0, 1)

Note

Median (lower quartile and upper quartile).

**TABLE 3:** Comparison of the length and frequency of hospitalization before and after introduction of CLZ

	Before introduction	After introduction	*P* value
Length of hospitalization of all patients (days)	157 (51, 348)	88 (10, 241)	<0.001**
Length of hospitalization of the long‐stay group (days)	684 (496.5, 730)	273 (124.5, 490)	<0.001**
Length of hospitalization of the non‐long‐stay group (days)	101 (36.25, 186.5)	64 (0, 139.5)	<0.001**
Frequency of hospitalization of all patients	2 (1, 3)	1 (0, 2)	<0.001**
Frequency of hospitalization of the long‐stay group	1 (0, 2)	0 (0, 2)	<0.001**
Frequency of hospitalization of the non‐long‐stay group	2 (1, 3)	1 (0, 1.75)	<0.001**

Note

Median (lower quartile, upper quartile).

**P < .01, *P < .05.

Both long‐stay and non‐long‐stay groups showed significant decreases in the length and frequency of hospitalizations after introduction (*P* < .001).

## 4 DISCUSSION

### 4.1 Treatment adherence rate and reasons for discontinuation

Inada, Oshibuchi, Ishigooka, and Nishimura reported the estimated 2‐year adherence rate of CLZ in Japan based on the Clozaril Patient Monitoring Service registry data. The continuation rates of CLZ treatment after all‐cause discontinuation were 78.2% and 72.9% at 1 and 2 years after treatment initiation, respectively.^4^ Our observational study showed similar results, with a high adherence rate even after 2 years. Regarding the group that discontinued, the majority of discontinuations occurred within 1 year after introduction, and most discontinuations occurred due to agranulocytosis or leukopenia during this period. The CGI‐S at discharge was significantly higher among those who discontinued treatment than among those who adhered to the CLZ treatment, indicating that some patients were refractory to CLZ. Among the CLZ‐refractory patients at our hospital, alternate treatment plans involving other antipsychotics and electroconvulsive therapy were attempted. However, treatment with alternate antipsychotics was not successful for some patients and they were reintroduced to CLZ. It has been reported that the length and frequency of hospitalizations decreased when CLZ was combined with other antipsychotics for CLZ‐refractory patients.^13^ In Japan, however, the use of CLZ in combination with other antipsychotics is prohibited in principle, as instructions in the package inserts of prescription drugs and the recommended treatment for CLZ‐refractory patients suggest CLZ combined with lamotrigine or modified electroconvulsive therapy (m‐ECT),^14^ m‐ECT combined with other antipsychotics,^15^ or the use of antipsychotics other than CLZ as a monotherapy or in combination with antipsychotics.^16^ However, as shown in overseas reports, reintroduction of CLZ, or a combination of CLZ with other antipsychotics, may be worth exploring for patients who discontinued CLZ for reasons other than agranulocytosis or leukopenia.

### 4.2 Decrease in the length and frequency of hospitalization

Our results showed that the introduction of CLZ significantly reduced the length and frequency of hospitalization. Decrease in the length and frequency of hospitalizations are often used as indicators in studies investigating the usefulness of CLZ. In Japan, Misawa, Suzuki, and Fujii investigated the length of hospitalization and degree of isolation during the 1st year after the introduction of CLZ.^17^ The authors reported that there was no significant decrease in the length of hospitalization, but there was a significant decrease in the frequency of isolation, thereby supporting the usefulness of CLZ. Our study reported different reductions as opposed to those reported by Misawa et al^17^ in the length of hospitalization before and after the introduction of CLZ. One of the reasons for this may be the larger sample size of this study. In contrast to the 35 participants in the study by Misawa et al^17^ our study had a total sample size of 233, with 55 participants in the long‐stay group alone, making it easier to show significant differences. In addition, the survey and evaluation periods of this study after the introduction of CLZ was 2 years, compared to 1 year in the study by Misawa et al^17^ Moreover, the evaluation period after the introduction of CLZ began at 18 weeks postintroduction in our study compared to 3 months postintroduction in Misawa et al^17^ which may have led to different results. As shown in Tables 2 and 3, reduction in the length of hospitalizations due to CLZ treatment tends to be more apparent over time, that is, more pronounced at 2 years than at 1 year. Improvement of psychiatric symptoms, such as improved communication and decreased impulsiveness, was observed from the early stages of introduction. However, it takes time for patients who are accustomed to an inpatient environment to acquire the desire to leave the hospital, and for their families and communities to prepare to accept patients who are in recovery. Therefore, if the observation period after the introduction of CLZ is short, the effect of reducing the length and frequency of hospitalization may not be detected.

### 4.3 CLZ treatment for the long‐stay group

There was no difference between the long‐stay group and the non‐long‐stay group in the duration or severity of illness before the introduction of CLZ, and both groups had similar adherence rates and improvements in CGI‐S. Significant differences between the long‐stay and non‐long‐stay groups were found in the duration of CLZ administration, CLZ dose at discharge, and length of hospital stay before discharge. The significant difference in duration of treatment reflects the priority given to the introduction of CLZ in the long‐stay group during hospitalization. Many patients in the long‐stay group had prolonged hospitalizations owing to lack of improvement with other antipsychotic treatments and were introduced to CLZ later, between 2010 and 2013. On the other hand, many patients in the non‐long‐stay group were admitted to our hospital after that time and were introduced to the CLZ sooner, which may have reflected in the difference in the duration of treatment. Differences in dosage and the length of hospital stay may be influenced by nonmedical factors such as postdischarge family and community acceptance and economic base, but these were not clarified in this study. However, it took twice as long for the long‐stay group to be discharged from the hospital compared to that for the non‐long‐stay group, suggesting that the effect of treatment emerged more slowly in the long‐stay group than in the non‐long‐stay group, even with the same severity of psychiatric symptoms. This illustrates that varied dosages and durations of treatment are needed to evaluate the effect of treatment on the long‐stay group. Thus, the treatment for the long‐stay group was more difficult than that for the non‐long‐stay group. However, the frequency of re‐hospitalization of the long‐stay group before and after the introduction decreased significantly, and a comparison of the number of rehospitalizations between the long‐stay and non‐long‐stay groups showed no significant difference, indicating that the same level of re‐hospitalization prevention effect can be expected in both the long‐stay and non‐long‐stay groups. Although some environmental factors can make discharge more difficult in the long‐term stay group than in the non‐long‐term stay group, such as the loss of a place to return and absence of a support person, the discharge rate in our hospital was not significantly different between the groups. This may be due to the fact that the community collaboration (day care and home nursing) that supports patients after discharge may be effective in promoting discharge and preventing readmission, but this is unclear as it was not the subject of this study.

### 4.4 Significance of observational studies

There are many reports of observational studies and cohort studies on the usefulness of CLZ for TRS.^18^, ^19^, ^20^, ^21^ However, recent meta‐analyses based on RCTs have cast doubt on the superiority of CLZ compared with other second‐generation antipsychotics.^9^ It is important to note that RCT‐based meta‐analyses have more reliable data than observational or cohort studies, such as the one we conducted here, and that their results often show no benefit of CLZ over other second‐generation antipsychotics. However, in RCTs involving CLZ, participants may only include a subset of the patient population with clinical symptoms requiring CLZ. In other words, it has been pointed out that the problem is that only patients who meet the TRS criteria but have the capacity to consent to participation in clinical trials are eligible for the trial.^17^ Patients who have been hospitalized for a long period in psychiatric hospitals in Japan, such as the long‐stay group, cannot be discharged despite the use of various antipsychotic drugs other than CLZ, either as single agents or in combination, and new treatment methods are required. Conducting an RCT with such a patient population faces difficulties in securing participants in terms of their ability to consent, and also in tracking their progress over time in terms of the length and frequency of hospitalizations. Therefore, while referring to the findings of RCTs, the results of retrospective observational studies such as the present study may be meaningful for the evaluation of the efficacy of CLZ in the long‐stay group.

### 4.5 Towards increased use of CLZ in future

Over 10 years have passed since CLZ became available in Japan, and the accumulated knowledge to date has revealed that the frequency of leukocyte count decline is comparable to that of overseas countries and that over 90% of cases occur within 1‐year postintroduction. Operating procedures for patients who had been on CLZ for 52 weeks were revised such that, in principle, monitoring of the white blood cell count once every 4 weeks is sufficient. In Okinawa^22^ and other prefectures, efforts are underway to enable and mobilize medical institutions that are concerned about the introduction of CLZ to provide treatment in the maintenance phase. Measures to reduce the burden on medical institutions that oversee introduction by separating such institutions from those that oversee treatment in the maintenance phase may help in furthering the use of CLZ. Of the TRS patients eligible for CLZ, the long‐stay group was more frequently admitted to the chronic care ward of psychiatric hospitals. Our study found a reduction in the duration of hospitalizations in the long‐stay group, indicating that CLZ is an effective treatment for the long‐stay group. As a result, our hospital has restructured its organization by downsizing the chronic care ward, expanding the emergency psychiatric ward, and reallocating staff to home nursing services, as previously reported.^13^, ^23^ However, it is difficult for many private psychiatric hospitals to conduct emergency psychiatric services and restructure their organizations, and financial support to reorganize chronic care wards may be necessary in order to encourage the use of CLZ.

CLZ is known worldwide as a treatment for TRS, and while its use is reported to be increasing, it is still underutilized in many countries. Bachmann et al^24^ analyzed data from 17 countries and found that the rate of CLZ use was 189.2 per 100 000 in Finland and 116.3 in New Zealand, compared with 42 in Italy, 14 in the USA, and 0.6 in Japan. Assuming an estimated proportion of patients with TRS of 20%–30% and a prevalence of schizophrenia of 0.7%–0.8%, the CLZ use rate should be between 140 and 240 per 100 000. In Japan, there are few reports on the use of CLZ in schizophrenic patients who have been hospitalized for more than a year and whose treatment has stagnated. In this study, we used CLZ in patients with prolonged hospitalization and confirmed its effectiveness in reducing hospitalization days and preventing readmission. However, in most countries, the use of CLZ is limited to a subset of patients with presumed TRS. The findings of this study indicate that the use of CLZ should be expanded. Further research is required to determine the disadvantages of schizophrenic patients who need CLZ but lack access to it.

### 4.6 Limitations

The limitations of this study include the fact that it was a retrospective observational study of patients treated at a single medical facility, as opposed to a study, which was designed with a comparison group. Additionally, 30% of the patients were transferred to another facility or completed CLZ treatment during the study period. This study examined the effect of clozapine; as the study included a clozapine discontinuation group, there may have been effects of drugs other than clozapine, and the hospitalization period and frequency after introduction may have increased. In addition, patients who have been transferred to other hospitals are excluded because the subsequent progress is unknown. Therefore, in future, multifacility research is desirable.　These limitations must be considered when interpreting the results of the present study.

## 5 CONCLUSION

The present study is a long‐term observational study of CLZ use in a private single‐specialty psychiatric hospital that has continued CLZ treatment, from introduction to maintenance. The results showed that the introduction of CLZ was effective in shortening the hospitalization period and preventing re‐hospitalization among long‐stay patients for whom discharge from the hospital was difficult owing to prolonged psychiatric symptoms and poor response to conventional antipsychotics. However, difficulties such as requiring more time for response to treatment to be observed among long‐stay patients compared to non‐long‐stay patients with TRS were also highlighted. In future, we will investigate the reason why the hospitalization period of the long‐stay group is extended, using a multifacility research approach.

## CONFLICT OF INTEREST

Kentaro Mizuno received lecture fees from Janssen Pharmaceutical, Otsuka Pharmaceutical, and Novartis Japan. All other authors declare no conflict of interest.

## AUTHOR CONTRIBUTIONS

KM conceptualized and finalized the manuscript. K.M and AS collected the data and performed statistical analysis. EM created the figures and tables. TS provided critical comments regarding the manuscript. All authors contributed to manuscript revision and have read and approved the submitted version.

## APPROVAL OF THE RESEARCH PROTOCOL BY AN INSTITUTIONAL REVIEW BOARD

This study was approved by the ethics committee of the Wakakusa Hospital (approval number 2020‐001).

## INFORMED CONSENT

Information regarding the study was posted in the hospital along with an explanation of how to request exclusion. Patients were provided with an adequate period of time to opt out. Patients who chose to not opt out were considered to have provided their informed consent to participate. Data were anonymized such that individuals could not be identified.

## Data Availability

The data that support the findings of this study are openly available on Figshare at https://figshare.com/s/0d21dff4c64da39cf27.
